# Supplementary material for: Differing Escape Responses of the Marine Bacterium Marinobacter adhaerens in the Presence of Planktonic vs. Surface-Associated Protist Grazers
Source: Int J Mol Sci. 2022 Sep 3;23(17):10082. doi: 10.3390/ijms231710082 (PMC9456119; doi:10.3390/ijms231710082)
Supplement: Supplementary file 1 [file ijms-23-10082-s001.zip › ijms-1862346-supplementary.pdf]

Section SA: Presence of different planktonic predator subpopulations in water and on surfaces.

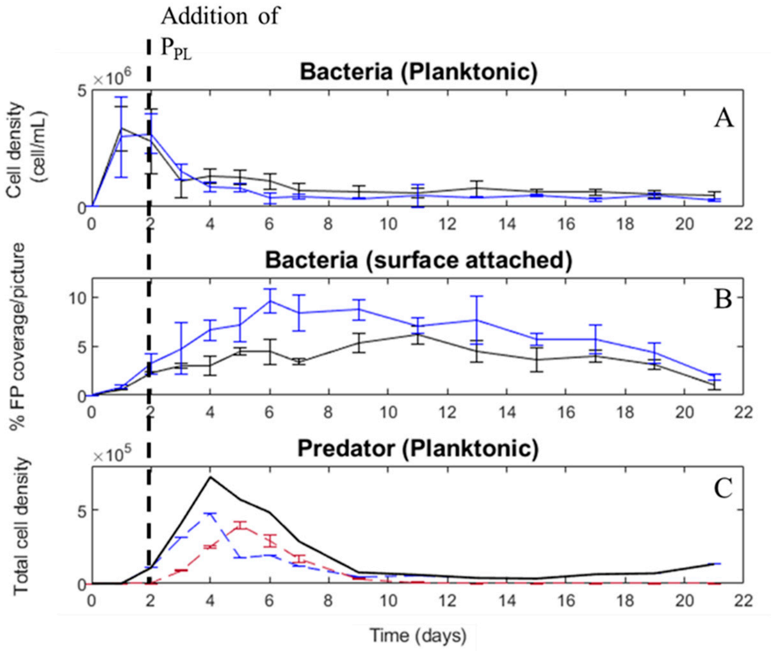

Figure S1. Predator-prey dynamics comparing the planktonic predation treatment vs. the control treatment. In Panel A and B, light blue lines represent the bacterial cell density in FL predation treatment in: water (Panel A) and attached to surfaces (Panel B), both in the presence of *C. roenbergensis*. Black lines represent bacteria in the control treatment without any protozoan predation, free-living bacteria (Panel A) and surface attached bacteria (Panel B). Panel C represents i) *C. roenbergensis* total cell density (black solid line) in the planktonic predation treatment, ii) of the surface attached subpopulation (brown dashed line) and iii) of the planktonic subpopulation (blue dashed line).

Section SB: Differences in bacterial attachment to surfaces in control, PSA treatment and PPL treatments.

Control treatment

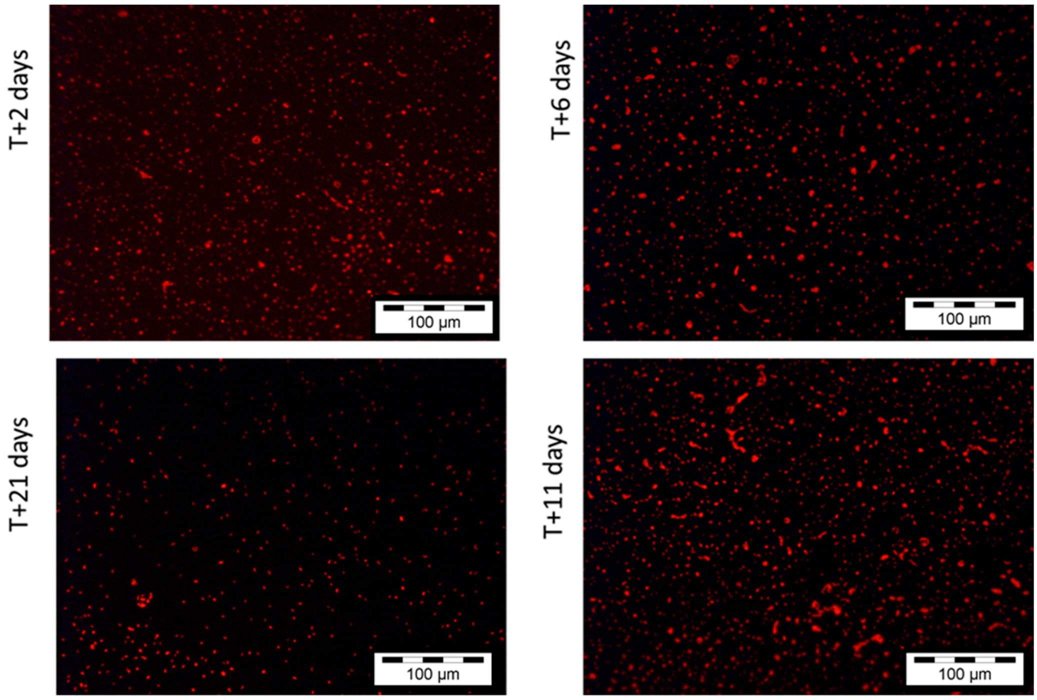

Figure S2. Bacterial numbers on the slide surface using the DSRed fluorescence protein. Photos were taken using a Leica epifluorescence microscope.

Planktonic predator PPL treatment

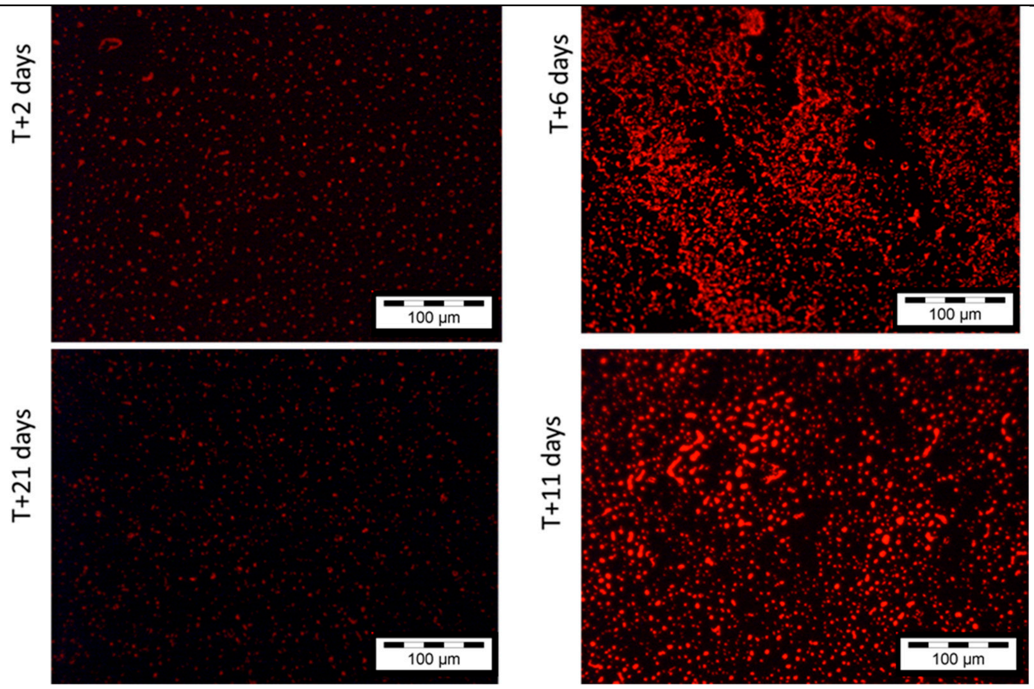

Figure S3. Bacterial abundance on the slide surface using the DSRed fluorescence protein. Photos were taken using a Leica epifluorescence microscope.

<u>Surface attached predator PSA treatment</u>

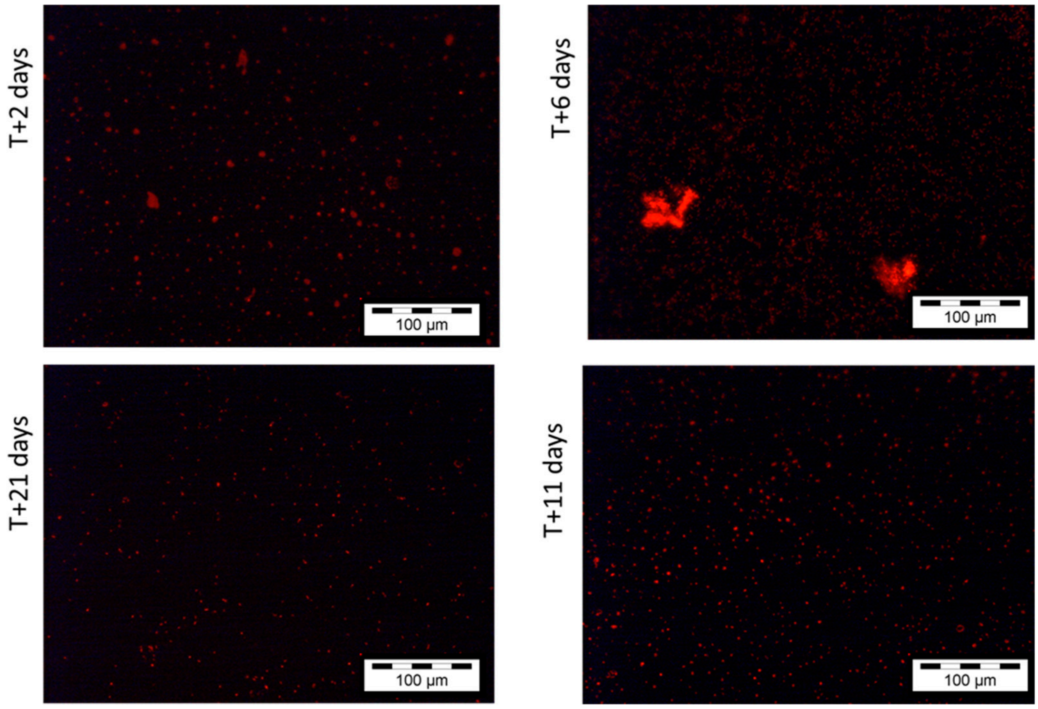

Figure S4. Bacterial biomass on the slide surface using the DSRed fluorescence protein. Photos were taken using a Leica epiflurescence microscope

Section SC: Clump formation after the addition of the planktonic predator (PPL) and Surface attached predator (PSA)

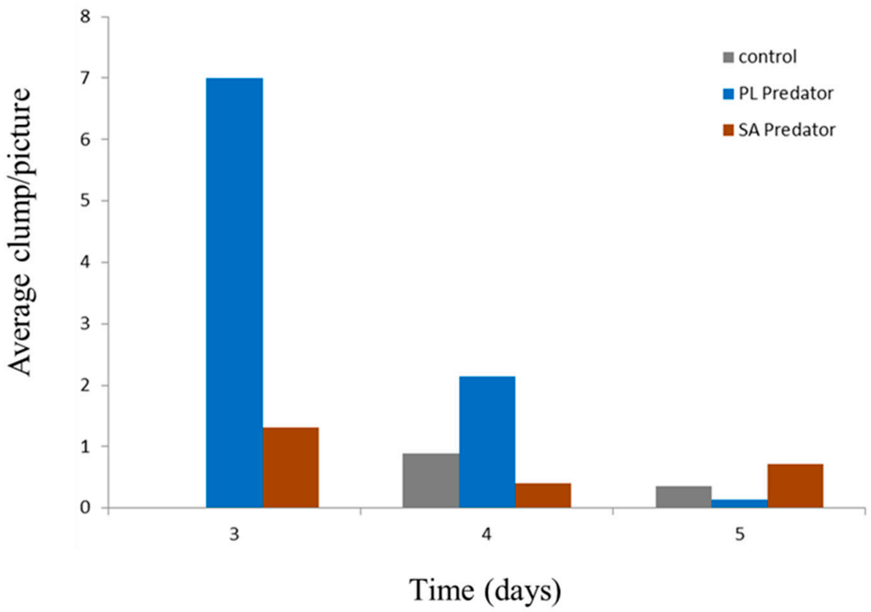

Figure S5. Clump number per 40× picture after the addition of the predators (day 2), compared to the control treatment. Clumps were considered when they were big to be that they were developed in order to avoid grazing.

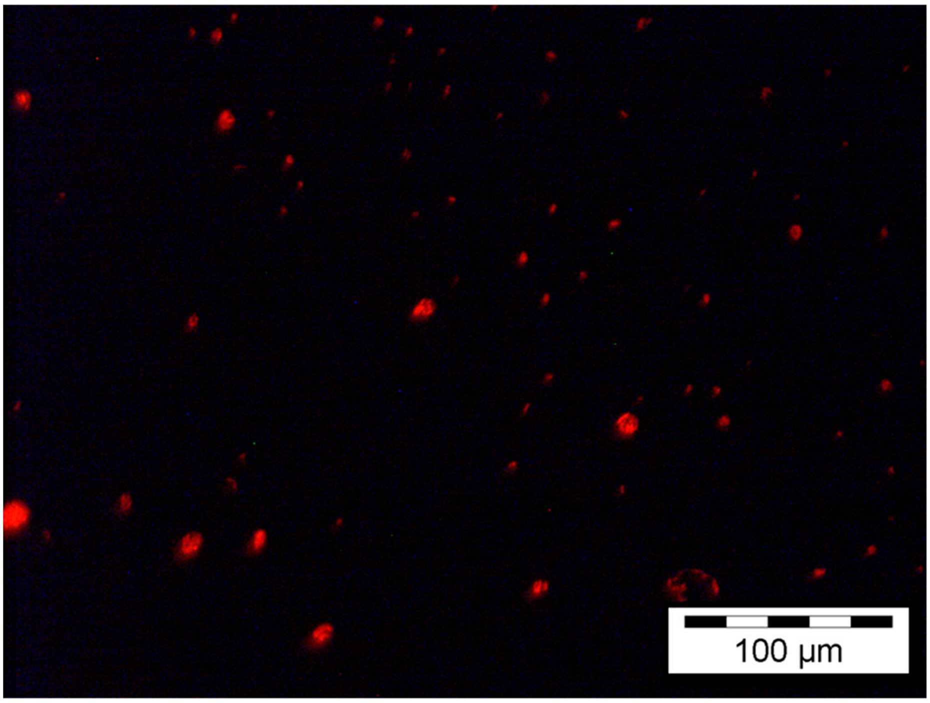

Figure S6. Clump formation in the PPL treatment on day 3, where most of the planktonic bacteria are forming aggregates in order to defend from predation by *Cafeteria roenbergensis*. Picture corresponds to 40× magnification using epifluorescence microscope Leica.

Section SD: Behavior of *M. adhaerens* Wild type (WT), chemotaxis (ΔCheA) and flagella (ΔFliC) knockout mutants, in the absence of any surface for attachment.

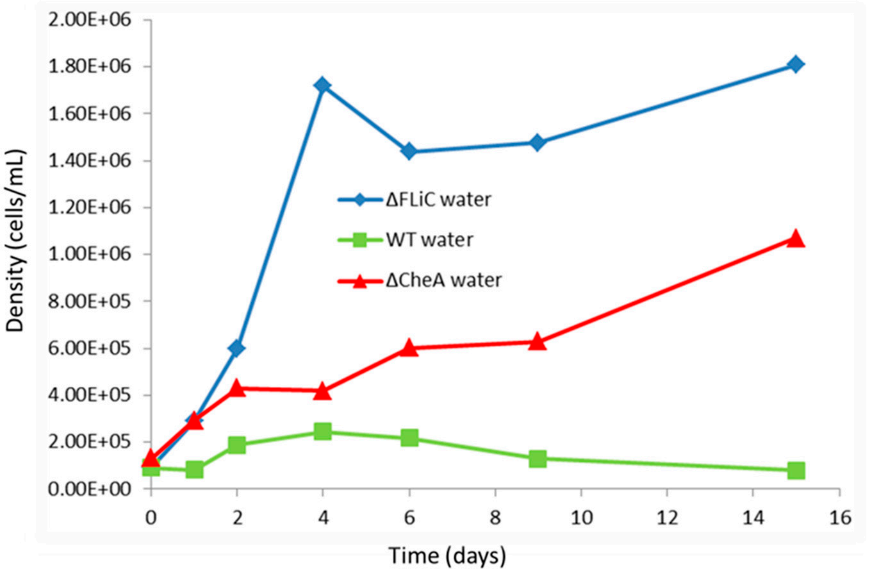

Figure S7. Growth rates of the different *M. adhaerens* strains in the water habitat without any surfaces neither grazers. We used the same nutrient in the water conditions than in the main experiment.

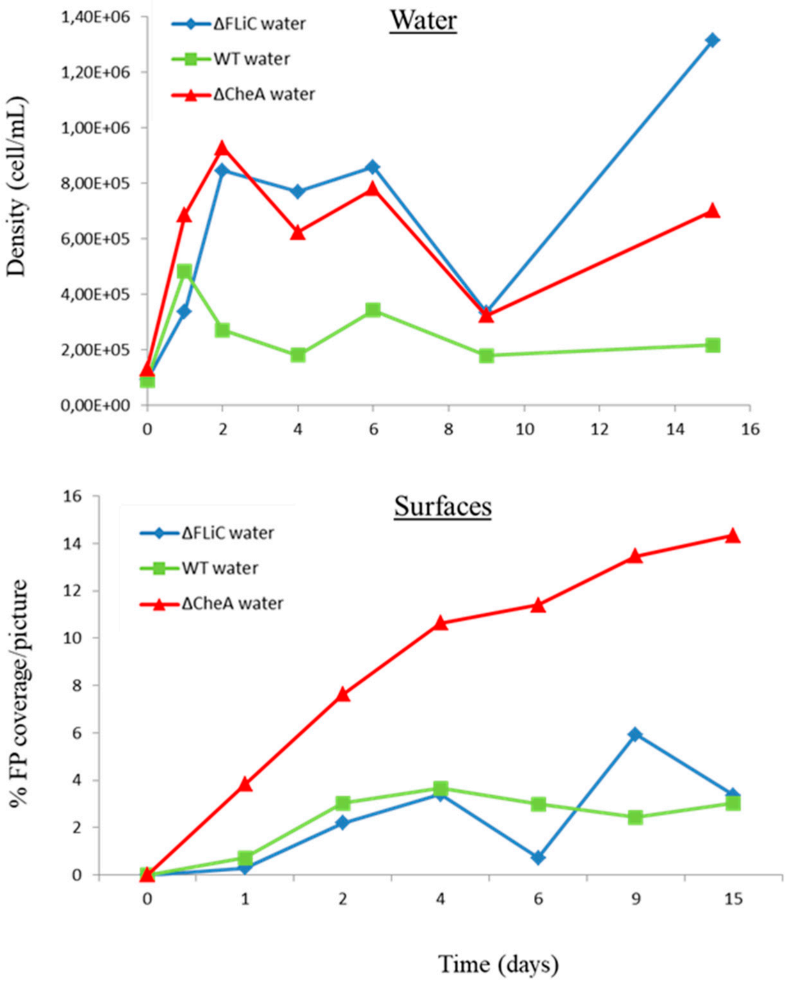

Figure S8. Growth rates for the different *M. adhaerens* strains in the water and surface compartment. Unlike in the experiment, surfaces have the same limiting nutrient concentrations than water, thus we made normal enriched particles (not enriched).

Section SE: Nutrient dynamics

Nutrient dynamics in the medium based on the evolution of the nitrogen compounds (nitrate, nitrite and ammonium). The limited nutrient in the cultures, ammonium (NH_4^+), shows a decrease in the first day and an additional increase after the 4th day in the case of the control treatment with only bacteria and after the 7th day in the predator treatments. Nitrite dynamics shows a very small concentration throughout the entire experiment. Nitrate shows a sharp increase during the first days of the experiment.

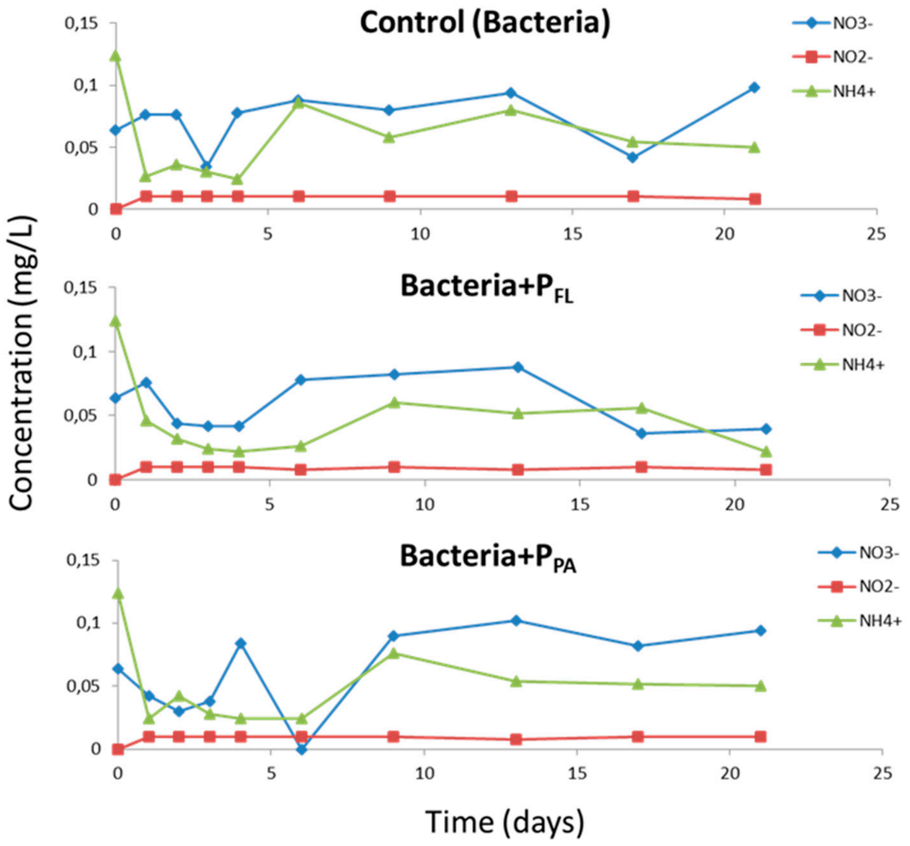

Figure S9: Nutrient dynamics of ammonium (green line), nitrate (blue line) and nitrite (red line) in the medium over time for the three different treatments.

Section SF: Approximate temporal and spatial ammonium leaching based on theoretical calculations.

Framework of ammonium leaching

Our calculation on ammonium leaching time from particles surfaces (200 µM) into the ambient water (2 µM) is calculated using the Fick's first law, where the coefficient of diffusion (J) is proportional to the diffusivity (DW) of ammonium in water at 20ºC and a negative concentration gradient between particle surfaces and water (See Eq. F1). We aimed to study the leaching time of ammonium as it is the limiting nutrient in our experimental approach. All values used for the calculation are given in Table F1.

$$J = -D_w \frac{dC}{dr}$$

Eq. F1

Our calculations show that the diffusive flux (J) is approximately 392 nmol cm-2 h-1. The total agar particle surface area for nutrient leaching is 10, 4 cm2, by multiplying both values, we are capable to calculate the total leakage from a single slide towards the water, i.e. 4 µmol slide-1 h-1. Based on our agar particle surface (with 1,2 mL of 200 µM of NH4+, we calculated the total NH4+ concentration present in a single agar slide, i.e. 0,24 µmol (Table S1). Thus, the total NH4+ concentration of a slide might be leached in less than one hour.

Approximate ammonium leaching profile from the surface into the water

The approximate profile indicates where the leaching process enriches the water and where more nutrients should be accessible for the bacteria. Our calculation show that ammonium in the water reaches approx. 0.5 mm from the center of each agar surface before the equilibrium between water and surface has been reached.

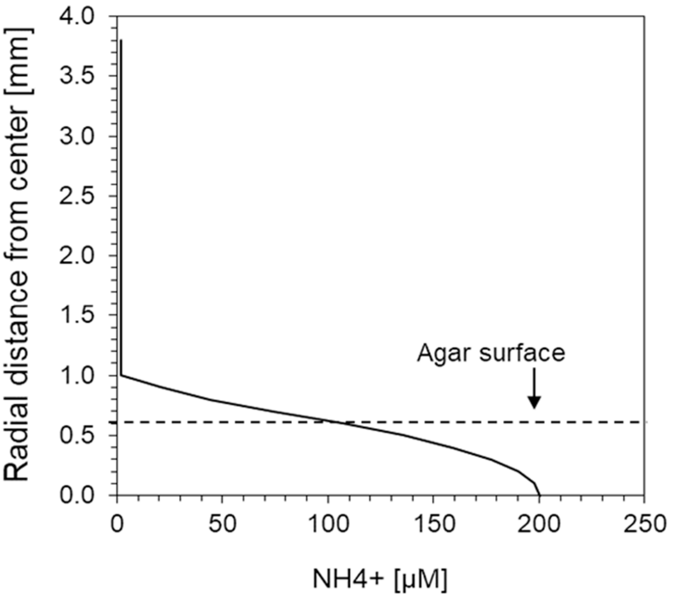

Figure S10. Approximate leaching profile for ammonium from each surface into the water. Ammonium concentration is 200 µM (agar) to 2 µM (in the ambient water). We also consider turbulence with a shear rate of 0.25 s^{-1}.

**Concentrations of NH4+ in ambient water (AW)**			
Parameter	Value	Units	Description
C (AW-NH_4^+)	2	µmol L^{-1}	concentration ammonium
V (AW)	200	mL	Volume ambient water in the batch cultures
Mol (AW)	0.4	µmol	mol ammonium cultures ambient water
**Concentrations NH4+ agar slides (Particle surfaces)**			
Parameter	Value	Units	Description
C (agar-NH_4^+)	200	µmol L^{-1}	concentration ammonium in the solution
V (agar)	1.2	mL	Volume agar solution (4x1.2 mL)
Mol NH_4^+/slide (agar)	0.24	µmol	mol ammonium in each agar volume
Total mol NH_4^+(agar)	0,96	µmol	mol ammonium in total agar volume (4x1.2 mL)
**Total NH4+ concentration (ambient water+ agar surfaces)**			
C (agar + ambient)	6.8	µmol L^{-1}	
**Diffusive Flux**			
Parameter	Value	Units	Description
Diffusioncoefficient (NH_4^+)	$1,65.10^{-5}$	$cm^2\ s^{-1}$	Value for NH_4^+ diffusivity in water at 20ºC
C (AW-NH_4^+)	2	µM	Ammonium conc. in the ambient water
C (agar-NH_4^+)	200	µM	Ammonium conc. in the particle surface
dC	-198	$nmol cm^{-3}$	Differences between ambient water and agar surfaces NH_4^+ conc.
dr	0.03	cm	Assumed to be the effective diffusive boundary layer DBL(eff)
**DIMENSIONS: Agar particle surfaces**			
Parameter	Value	Units	Description
l-length	3.5	cm	length of the microscope slide where the solid agar solution was pored
w-width	2.5	cm	Width of the microscope slides
h-height	0.14	cm	Height of the agar solution poured into the slides
Volume in each slide	1.20	cm^3	Total volume of the solution pored per slide
Surface area (total)	19.1	cm^2	The total surface are is calculated as A=2wl + 2lh + 2hw
Surface area (for diffusion)	10.4	cm^2	since one large surface area is covered by glass slide -> no diffusion possible

Table S1. Parameter values used for the calculation of the diffusion coefficient.

Section SG: Use of ImageJ for calculating bacterial number on particle surfaces.

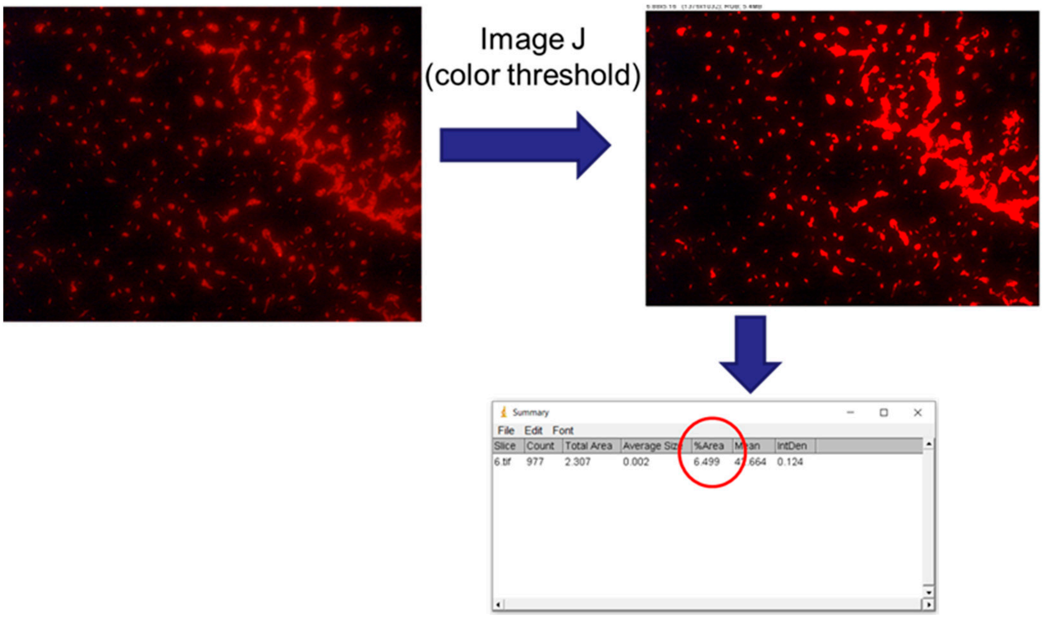

Section SH: Additional information

H.1. Experimental

An important methodological point to be discussed, which potentially interferes with our observations, is the detection method used to count *M. adhaerens* throughout time. The presence of a plasmid that encodes a DSRed fluorescence protein makes it easy to detect and count the bacterial cells, but may lead to counting biases. Especially under low nutrient conditions, lower expression of the DSRed fluorescence protein may lead to fading or even total loss of fluorescence making cell counts less reliable. This problem is increasing over time as organic matter and nutrients are increasingly depleted over time as bacterial cells often respond to starvation by reducing cell size and gene expression [41]. To overcome this problem we have determined the fraction of DSRed-stained bacterial cells of total cells (counted after DAPI staining) under decreasing nutrient concentrations and corrected our cell counts accordingly.

The presence of possible bacterial background contamination could also not being discarded. To reduce this possibility the predators were treated with antibiotic and before starting the experiments the presence of bacteria in the water (Planktonic predator) and agar (Surface-attached predator) were tested by growing part of both cultures in MB medium at 37 ºC. We made sure that bacteria from predator cultures was not growing at least in 2 days after setting an incubation in 15 mL falcon tubes using MB medium at 37ºC. If there is bacterial contamination we could see a bacterial cloud in the 15 mL falcon tube with 50 µL of the flagellate culture plus 3 mL MB medium. For background contamination in amoebae we introduced and agar block into 3 mL MB medium and incubate it at 37°C. The absence of bacterial cloud in the first 2 days of incubation meant that background contamination was low. Additionally, we added the predators 2 days after the initial colonization of bacteria that in our oligotrophic conditions might have uptaken most resources reducing the risk of the bacterial contamination outweighed the target bacteria *M. adhaerens*.

Furthermore, nutrient concentrations were two orders of magnitude (100 times) higher on the solid surfaces than in the water, which may have led to an initial leaching event of dissolved nutrients, in particular ammonium, from the surfaces into the water. According to our measurements it reaches equilibrium in less than one hour (see Section SF). This is supported by our theoretical calculations based on the Fick's first Law and the porosity of agar as well as the differences in ammonium concentration in agar and water. These theoretical calculations revealed that the equilibrium concentration between the two habitats is indeed reached in < 1 hour (see Section SF). Thus, we assume that leaching just plays a role during the initial part of the experiment. As we have added the grazers only at day 2 of the experiment, the leaching effect should be negligible. In addition, the nutrient profile shows an increase in ammonium concentration in the 0.5 mm zone surrounding the agar surface (See Section SF) indicating that nutrient concentrations remained high at the solid surface, but low in the water outside of the diffusion gradient above the enriched agar surface.

H.2. GAM analysis

Our statistical analysis explained the dynamics of the surface attached bacteria quite well (R = 0.837, Deviance explained = 89.6%). However, the high complexity of defensive responses may explain the observed large residuals (see Section SG) for the dynamics of planktonic bacteria (Fig S1, R = 0.579, Deviance explained = 64%), as the model did not include, e.g., bacterial clumping as an active defense trait against planktonic predators. Another possibility, as anticipated above, could be the growth/settling of bacteria on the beaker walls that were not included in the counts of surface-attached bacteria. Attach-

ment of the planktonic protists to the available surfaces could also impact these residuals, even when literature (e.g., [21]) assumes otherwise.

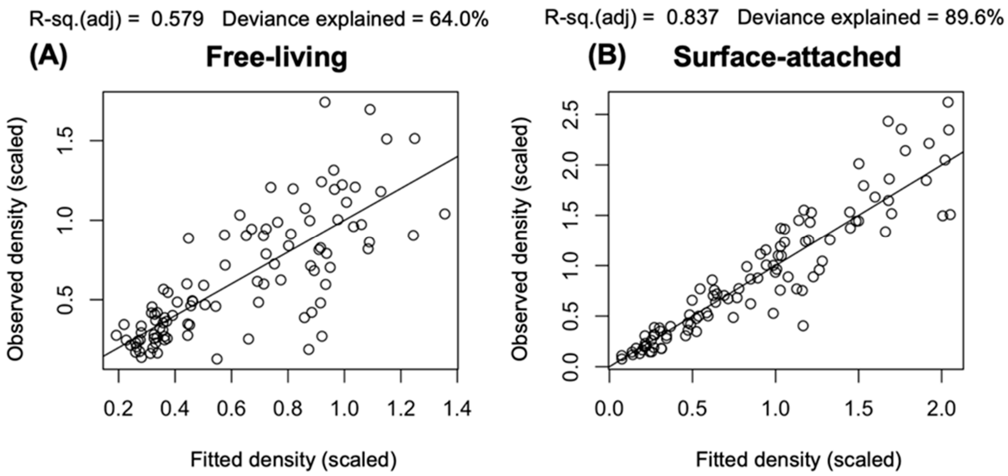

Figure S11. Predicted vs. observed values of the scaled density of planktonic free-living (A), and surface-attached (B) *M. adhaerens* (ΔCheA). Black lines mean the prediction corresponds to the model.
